# Instant messaging-delivered brief motivational interviewing for noncommunicable disease patients with no intention to quit smoking

**DOI:** 10.1038/s41746-026-02578-6

**Published:** 2026-04-11

**Authors:** Laurie Long Kwan Ho, Dorothy Ngo Sheung Chan, Joyce Oi Kwan Chung, Kai Chow Choi, Wei Xia, William Ho Cheung Li

**Affiliations:** 1 The Nethersole School of Nursing, The Chinese University of Hong Kong, Hong Kong SAR, China; 2 School of Nursing, The University of Hong Kong, Hong Kong SAR, China; 3 School of Nursing, The Hong Kong Polytechnic University, Hong Kong SAR, China; 4 School of Nursing, Sun Yat-Sen University, Guangdong, China

**Keywords:** Diseases, Health care, Medical research

## Abstract

Most noncommunicable disease patients who continue smoking are chronic users with no intention to quit. This multicentre randomised controlled trial evaluated the effectiveness of brief motivational interviewing (MI) delivered using mobile instant messaging on smoking abstinence in this population. Overall, 728 adults were recruited and randomised (1:1) to the intervention or control group. Intervention group received a face-to-face brief MI session followed by instant messaging-delivered brief MI for 6 months. Control group received a face-to-face health advice consultation and a self-help booklet. Follow-ups were conducted at 3, 6, and 12 months. The primary outcome was biochemically validated smoking abstinence at 12 months, defined as self-reported abstinence from smoking in the last 7 days confirmed by exhaled carbon monoxide <4 ppm and saliva cotinine <15 ng/mL. At 12 months, 4.1% of participants in the intervention group met the primary outcome, compared with 1.4% in the control group (rate ratio [RR], 3.00; 95% confidence interval [CI], 1.10–8.17; *P* = 0.03). Secondary outcomes were also significantly higher in the intervention group, including validated abstinence at 6 months (4.7% vs 1.4%; RR, 3.40; 95% CI, 1.27–9.12; *P* = 0.02) and intention to quit at both 3 months (30.8% vs 18.9%; RR, 1.63; 95% CI, 1.25–2.13; P < 0.001) and 6 months (33.7% vs 24.1%; RR, 1.40; 95% CI, 1.10–1.79; *P* = 0.01). The intervention could be easily integrated into existing smoking cessation services, effectively motivating individuals who had no intention of quitting to quit—addressing one of the major challenges in tobacco control.

## Introduction

The World Health Organisation (WHO) highlighted tobacco use as a major cause of death and disability resulting from noncommunicable diseases (NCDs)^1^. Continued smoking among NCD patients has further detrimental effects on health and treatment outcomes, increasing the risks of mortality and disease progression and decreasing treatment efficacy^2,3^. However, previous studies have shown that being diagnosed with an NCD or requiring medical attention for an NCD may not necessarily increase the likelihood of an individual quitting smoking^4,5^. We previously found that most NCD patients who continue to smoke are heavy chronic users with no intention to quit^6–8^, implying additional challenges in reaching or engaging these individuals. Our systematic review of 10 studies, including non-randomised and randomised clinical trials (RCTs), aimed at helping adults with NCDs quit, identified major methodological flaws and highly heterogeneous intervention content^7^. These trials may also have been susceptible to selection bias by excluding individuals with no intention to quit, despite this group representing a substantial proportion of those living with NCDs^6,7,9^. Thus, more efforts need to be directed toward this subset of people who smoke.

The increasing ubiquity of mobile instant messaging tools could be a solution, offering a promising avenue to the success of attempts to abstain from smoking, especially among hard-to-reach individuals^10,11^. Such groups are typically those who are not adequately reached by mainstream healthcare services due to system‑level barriers and shortcomings, including the healthcare system’s limited capacity to reach, adapt, and deliver services that are accessible and acceptable to diverse populations^12^. Mobile messaging approaches may help address these gaps by providing flexible, low-threshold, and scalable support that complements existing tobacco control measures. In Hong Kong, 96.4% of residents own a smartphone, and more than 90% of them frequently use mobile instant messaging tools^13^. However, a local population-based survey found that only 1.5% of individuals who smoke had tried smoking cessation services delivered by mobile technology^14^. Our qualitative study found that individuals with NCDs who smoke were considerably ambivalent between changing other disease-related unhealthy behaviours to control their NCDs or quitting smoking, unaware of the association between NCDs and smoking^15^. To address the issues of individuals with NDCs who have no intention of quitting and are experiencing considerable ambivalence about quitting, specific counselling techniques and professional clinical judgment are required in addition to mobile technology^16,17^.

We developed an intervention model that delivers brief motivational interviewing (MI) using the foot-in-the-door technique, which allows individuals who smoke to choose an unhealthy behaviour for modification as a first step to encourage their engagement in the intervention, with the goal of increasing the likelihood of compliance with a larger request once an individual accedes to the smaller request (Supplementary Fig. 1)^18^. The foot-in-the-door technique may also help to attract individuals who have no intention of quitting and increase their compliance. MI is a directive, patient-centred counselling approach that is widely used to elicit addictive behaviour change^19^. It served to encourage individuals who smoke to explore and resolve possible ambivalence, strengthening their intrinsic motivation and competence to achieve behavioural change^19,20^. A meta-analysis of 31 RCTs found traditional MI ineffective in promoting smoking cessation among individuals with NCDs who smoke^21^. While there is no definitive evidence attributing this outcome to the intensive intervention design^22^, existing literature suggests that the burden of multiple sessions may reduce recruitment and increase attrition rates due to heightened time and resource demands^23,24^. The inflexible nature of such delivery may elicit psychological reactance, particularly among individuals who smoke and have no intention of quitting^23^. Thus, mobile instant messaging-delivered brief MI, which utilises shorter and more flexible strategies, may be more effective in engaging smokers with NCDs who have no intention to quit^25,26^. We identified no trials examining the effectiveness of mobile instant messaging-delivered brief motivational interviewing for smoking cessation, nor any trials specially targeting individuals with NCDs who have no intention of quitting (search was updated on June 21, 2025). The current trial evaluated the effectiveness of a brief MI smoking cessation intervention delivered using mobile instant messaging tools on abstinence among individuals with NCDs who have no intention to quit.

## Results

### Recruitment and participants

**Fig. 1:**
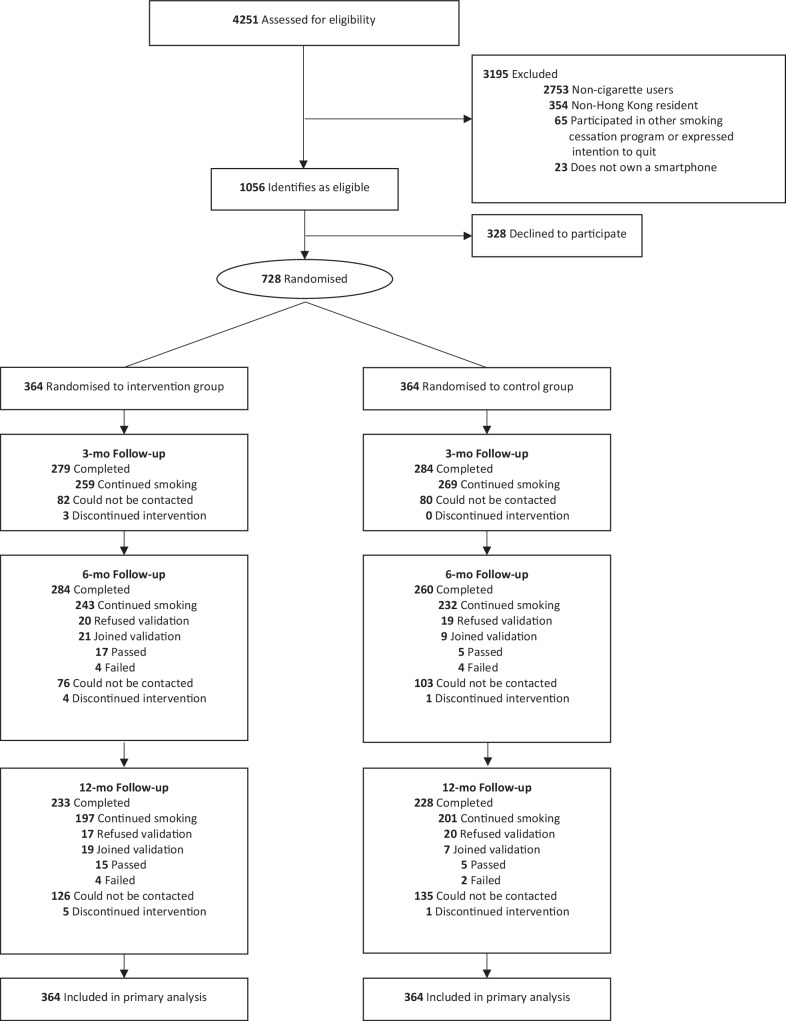
CONSORT Flow Diagram. The figure illustrates the participant recruitment and study flow.

From June 1, 2021 to February 28, 2022, 4,251 NCD patients were screened for eligibility. Of the 1,056 eligible individuals, 728 (68.9%) consented to participate and were randomised to the intervention (*n* = 364) and control (*n* = 364) groups (Fig. 1). The participants included 680 men and 48 women with a mean (standard deviation) age of 54.27 (12.58) years at baseline (Table 1). The two study groups had similar demographic and smoking characteristics. The retention rates were 77.3% (563/728), 74.7% (544/728), and 63.3% (461/728) at 3, 6, and 12 months, respectively. The baseline characteristics were comparable between those who completed the study and those who dropped out at 12 months (Supplementary Table 1). The biochemical validation tests were attended by 43.5% (30/69) and 41.3% (26/63) of the participants at 6 and 12 months, respectively, with the rates comparable between the two groups (all *P* > 0.05).

**Table 1:** Baseline Characteristics of the Participants (*N* = 728)

Characteristics	No. (%)^a^	
	Intervention (*n* = 364)	Control (*n* = 364)
Age, mean (SD), y	53.7 (13.4)	55.4 (12.1)
Sex		
Male	338 (92.9)	342 (94.0)
Female	26 (7.1)	22 (6.0)
Educational attainment		
Primary or below	72 (20.0)	93 (25.9)
Secondary	250 (69.4)	229 (63.8)
Tertiary	38 (10.6)	37 (10.3)
Employment status		
Employed	249 (68.4)	214 (58.8)
Unemployed	115 (31.6)	150 (41.2)
Diagnosis		
Cardiovascular diseases	78 (21.4)	74 (20.4)
Cancers	1 (0.3)	2 (0.6)
Chronic respiratory diseases	39 (10.7)	43 (11.8)
Diabetes	20 (5.5)	30 (8.3)
Multiple chronic conditions^b^	226 (62.1)	214 (59.0)
Years of smoking, mean (SD), y	32.6 (13.3)	34.4 (14.7)
Daily cigarette consumption		
1–10	121 (33.5)	101 (28.1)
11–20	120 (33.2)	140 (38.9)
21–30	91 (25.2)	98 (27.2)
>30	29 (8.0)	21 (5.8)
Nicotine dependence by the FTND^c^		
Mild, 0–3	48 (13.4)	39 (10.9)
Moderate, 4–5	112 (31.4)	126 (35.2)
Severe, 6–10	197 (55.2)	193 (53.9)
Previous quit attempts		
Yes (within 6 months)	123 (34.1)	102 (28.3)
Yes (beyond 6 months ago)	164 (45.4)	167 (46.4)
No	74 (20.5)	91 (25.3)
Selected unhealthy behaviours		
Unhealthy diet	114 (31.3)	125 (34.3)
Physical inactivity	106 (29.1)	110 (30.2)
Unhealthy sleep patterns	109 (29.9)	97 (26.6)
Excessive alcohol use	35 (9.6)	32 (8.8)

*FTND* Fagerström Test for Nicotine Dependence.

^a^ Sample sizes varied because of missing data on some variables.

^b^ Multiple chronic conditions are defined as two or more concurrent chronic diseases.

^c^ A six-item scale scored from 0 to 10, with higher scores indicating greater nicotine dependence.

### Outcomes

Biochemically validated smoking abstinence at 12 months (primary outcome; rate ratio, 3.00; 95% confidence interval [CI], 1.10–8.17; *P* = 0.03; Table 2) and 6 months (rate ratio, 3.40; 95% CI, 1.27–9.12; *P* = 0.02) were significantly higher in the intervention group than in the control group. However, self-reported 7-day point prevalence smoking abstinence (PPA) at all follow-up time points were comparable between the two groups (all *P* > 0.05).

**Table 2:** Primary and Secondary Outcomes

Outcome		No. (%)			
		Intervention (N = 364)	Control (N = 364)	Rate ratio^a^ (95% CI)	P value^b^
***Primary outcome***					
Biochemically validated smoking abstinence	6 months	17 (4.7)	5 (1.4)	3.40 (1.27–9.12)	0.02^b^
	12 months	15 (4.1)	5 (1.4)	3.00 (1.10–8.17)	0.03^b^
***Secondary outcomes***					
Self-reported 7-day PPA	3 months	20 (5.5)	15 (4.1)	1.33 (0.69–2.56)	0.39
	6 months	41 (11.3)	28 (7.7)	1.46 (0.93–2.32)	0.10
	12 months	36 (9.9)	27 (7.4)	1.33 (0.83–2.15)	0.24
Intention to quit^c^	3 months	106/344 (30.8)	66/349 (18.9)	1.63 (1.25–2.13)	<0.001^b^
	6 months	109/323 (33.7)	81/336 (24.1)	1.40 (1.10–1.79)	0.01^b^
	12 months	99/328 (30.2)	84/337 (24.9)	1.21 (0.95–1.55)	0.13
Self-reported smoking reduction ≥ 50%^c^	3 months	84/344 (24.4)	77/349 (22.1)	1.10 (0.84–1.45)	0.46
	6 months	143/323 (44.3)	100/336 (29.8)	1.49 (1.21–1.83)	<0.001^b^
	12 months	106/328 (32.3)	93/337 (27.6)	1.17 (0.93–1.48)	0.19
Quit attempts^c^	3 months	58/344 (16.9)	58/349 (16.6)	1.02 (0.73–1.41)	0.93
	6 months	95/323 (29.4)	48/336 (14.3)	2.06 (1.51–2.81)	<0.001^b^
	12 months	61/328 (18.6)	46/337 (13.6)	1.36 (0.96–1.94)	0.08
Self-reported changes in unhealthy behaviours chosen	3 months	138 (37.9)	84 (23.1)	1.64 (1.31–2.07)	<0.001^b^
	6 months	119 (32.7)	68 (18.7)	1.75 (1.35–2.27)	<0.001^b^
	12 months	74 (20.3)	56 (15.4)	1.32 (0.96–1.81)	0.08

*PPA* Point-prevalence abstinence.

^a^ Rate ratio was estimated by a generalised linear model using a log binomial link function with the control group as the reference.

^b^ Remained significant after Holm’s multiplicity adjustment.

^c^ Participants who self-reported smoking abstinence for at least seven days were excluded.

Excluding quitters, intention to quit at 3 months (rate ratio, 1.63; 95% CI, 1.25–2.13; *P* < 0.001) and 6 months (rate ratio, 1.40; 95% CI, 1.10–1.79; *P* = 0.01), self-reported smoking reduction ≥ 50% at 6 months (rate ratio, 1.49; 95% CI, 1.21–1.83; *P* < 0.001), and quit attempts at 6 months (rate ratio, 2.06; 95% CI, 1.51–2.81; *P* < 0.001) were significantly higher in the intervention group than in the control group.

Self-reported changes in the chosen unhealthy behaviours at 3 months (rate ratio, 1.64; 95% CI, 1.31–2.07; *P* < 0.001) and 6 months (rate ratio, 1.75; 95% CI, 1.35–2.27; *P* < 0.001) were significantly higher in the intervention group than in the control group.

As the baseline characteristics were comparable between those who completed the study and those who dropped out at 12 months, it is plausible that the missing data owing to dropout cases were MCAR. The sensitivity analyses of primary and secondary outcomes obtained by completer case analysis yielded comparable point estimates of the intervention effects obtained by ITT analysis (Table 3).

**Table 3:** Sensitivity Analyses of Primary and Secondary Outcomes based on Completer Case Analysis

Outcome		No. (%)			
		Intervention	Control	Rate ratio^a^ (95% CI)	*P* value^b^
***Primary outcome***					
Biochemically validated smoking abstinence	6 months	17 (8.2)	5 (2.5)	3.11 (1.17 – 8.32)	0.024^b^
	12 months	15 (6.4)	5 (2.2)	2.94 (1.09 – 7.94)	0.034^b^
***Secondary outcomes***					
Self-reported 7-day PPA	3 months	13 (6.5)	10 (5.1)	1.36 (0.71 – 2.60)	0.356
	6 months	29 (14.0)	21 (10.6)	1.34 (0.86 – 2.10)	0.202
	12 months	36 (15.5)	27 (11.8)	1.31 (0.82 – 2.08)	0.262
Intention to quit^c^	3 months	81 (43.3)	45 (24.2)	1.67 (1.29 – 2.15)	<0.001^b^
	6 months	77 (43.3)	64 (36.0)	1.29 (1.03 – 1.61)	0.029 ^b^
	12 months	99 (50.3)	84 (41.8)	1.20 (0.97 – 1.49)	0.092
Self-reported smoking reduction ≥ 50%^c^	3 months	65 (34.8)	51 (27.4)	1.13 (0.88 – 1.47)	0.343
	6 months	108 (60.7)	76 (42.7)	1.37 (1.14 – 1.64)	0.001^b^
	12 months	106 (53.8)	93 (46.3)	1.16 (0.96 – 1.42)	0.134
Quit attempts^c^	3 months	42 (22.5)	43 (23.1)	1.04 (0.75 – 1.43)	0.817
	6 months	68 (38.2)	40 (22.5)	1.89 (1.40 – 2.54)	<0.001^b^
	12 months	61 (31.0)	46 (22.9)	1.35 (0.97 – 1.88)	0.071
Self-reported changes in unfavorable behaviors chosen	3 months	98 (49.0)	61 (31.1)	1.67 (1.35 – 2.07)	<0.001^b^
	6 months	84 (40.6)	56 (28.3)	1.60 (1.25 – 2.04)	<0.001^b^
	12 months	74 (31.8)	56 (24.6)	1.29 (0.96 – 1.74)	0.088

*PPA* Point-prevalence abstinence.

^a^ Rate ratio was estimated by a generalised linear model using a log binomial link function with the control group as the reference.

^b^ Remained significant after Holm’s multiplicity adjustment.

^c^ Participants who self-reported smoking abstinence for at least seven days were excluded.

### Engagement analysis

In the intervention group, 50.8% of the participants (185/364) were deemed to be effectively engaged (i.e., had at least three conversations). The logistic regression analysis showed that the participants who were effectively engaged had significantly higher biochemically validated smoking abstinence at 12 months than those who were not effectively engaged (7.0% [13/185] vs 1.1% [2/179]; odds ratio, 6.69; 95% CI, 1.49–30.08; *P* = 0.013; Supplementary Table 2 & 3).

## Discussion

Although global smoking prevalence has significantly declined over the past few decades, the rate of decline has slowed due to the persistence of many individuals with no intention to quit^27^. One major gap in tobacco control is the lack of effective strategies to engage this subset of individuals in quitting smoking^7^, given that they are less amenable to current tobacco control interventions or policies and ultimately comprise an increasing proportion of the remaining individuals who smoke in the community^6,27^. The current study adopted a proactive approach (i.e., actively approaching individuals with NCDs who smoke) and integrated mobile instant messaging-delivered brief MI with the foot-in-the-door technique (i.e., allowing individuals to choose an unhealthy behaviour for modification as a first step), which was highly effective in recruiting individuals with NCDs who had no intention to quit. The intervention required participants to focus on a single self‑selected unhealthy behaviour. Although all participants had multiple concurrent unhealthy behaviours at baseline, prioritising the behaviour they most wished to change is consistent with MI principles, which emphasise autonomy, prioritisation, and attention to the behaviour for which an individual shows the greatest readiness to change. This approach also aligns with the foot‑in‑the‑door technique, in which commitment to a small, self‑initiated change can facilitate broader behaviour modification. This intervention design therefore does not limit generalisability to individuals with multiple behaviour change needs at baseline. In addition, although the principles and spirit of MI informed the intervention, the more adaptable and simpler brief MI approach was more acceptable to individuals with no intention to quit. The results showed that 68.9% of the eligible individuals consented to participate in the study, indicating a higher recruitment rate than those reported in other smoking cessation trials targeting NCD patients^6,8^. The current study also found higher retention rates at 3 months (77.3%) and 6 months (74.7%) than other smoking cessation trials, which typically had retention rates of approximately 65–70%^6,8^. The rate of effective engagement with the intervention (50.8%) was higher than those reported in other smoking cessation trials of mobile health interventions that included individuals with no intention to quit (16.8%)^11^.

Consistent with previous studies conducted in Hong Kong outpatient clinics, this study found that approximately 70% of the participants had smoked for more than 10 years and 86% had moderate-to-high nicotine dependency^6,8^. However, the lower biochemically validated quit rates in this study (versus those in other smoking cessation trials^6,8,11^) may be due to the study’s focus on individuals with no intention to quit. Nevertheless, rather than quitting immediately, a sizeable portion of our participants became intended to quit or had started reducing their cigarette consumption at the 3- and 6-month follow-ups.

This RCT showed that mobile instant messaging-delivered brief MI was more effective than a generic health advice consultation and a self-help smoking cessation booklet, significantly increasing the biochemically validated quit rate (tripled at 6 and 12 months) and promoting other smoking cessation outcomes among individuals with NCDs who had no intention of quitting. However, no significant differences in self-reported 7-day PPA at 6 and 12 months were observed between the intervention and control groups, indicating that this result may have been influenced by social desirability bias or recall bias^28^. The biochemical validation tests were necessary to verify self-reported smoking abstinence and prevent potential bias resulting in overestimation or underestimation of the intervention effect^29^. The real-world effect of the intervention would probably be stronger because the control group in this study received a generic health advice consultation, which is not readily available in clinical settings.

This trial has some limitations. First, a sizeable proportion of participants lost to follow-up may have introduced a non-response bias. However, the results of sensitivity analyses obtained by completer case analysis yielded point estimates that were comparable to those obtained by ITT analysis. Second, the study measured 7-day PPA, a single‑time‑point measure that captures abstinence only during the 7 days immediately preceding assessment. Although widely used in smoking cessation research, PPA may overestimate long‑term abstinence compared with continuous abstinence because it does not account for intermittent lapses across the full follow‑up period, a limitation that may be particularly relevant in behavioural interventions^30^. Despite incorporating biochemical validation to enhance the objectivity of abstinence assessment, future research could, where feasible, employ more robust measures such as continuous abstinence to better evaluate the sustainability of intervention effects. Third, the participation rates for biochemical validation tests were less than 50%, despite the rates being comparable between the two study groups. The study, however, had similar participation rates to those in previous studies conducted in outpatient clinics^6,8^. Fourth, the intervention specifically targeted individuals with NCDs who were more likely to smoke heavily than general populations and may have been likely to reject or avoid compliance with smoking cessation interventions^6,7,9^. Further trials may be needed to apply the study findings to individuals who smoke with other characteristics. Fifth, the study focused on comparing event rates at discrete time points rather than using longitudinal models such as generalised estimating equations. While this approach aligns with the study objective, it limits the ability to infer within‑person change over time and may result in reduced statistical efficiency compared with longitudinal methods, even with multiplicity adjustment applied. Sixth, the intervention limited participation to individuals with smartphones and the ability to use mobile instant messaging tools. However, identifying and comparing the potential differences in the baseline characteristics between smartphone users and non-users was beyond the study’s scope. In future applications, SMS could serve as an alternative communication channel for individuals who smoke but lack access to mobile instant messaging platforms.

In this study, over 99% of the NCD patients screened were smartphone users and were able to use mobile instant messaging tools. The widespread use of mobile instant messaging tools offers a new channel for healthcare professionals to easily provide resources that individuals with no intention to quit may find acceptable. It is understandable; participants in this study received brief MI messages without committing to quitting at the baseline, reducing their psychological reactance toward the intervention. This mode of delivery requires limited resources and is adaptable and facilitates interventions for closely monitoring patients’ progress. Although the first brief MI sessions were conducted face-to-face, establishing rapport with participants at baseline is essential for enhancing engagement with the intervention—particularly among those who had no intention of quitting at the outset. Each session lasted only 10 min, making it feasible for implementation by healthcare professionals with minimal resources. Therefore, the current intervention model could be easily integrated into existing smoking cessation services, encouraging individuals who had no intention of quitting to quit. Its effectiveness merits further trials in real-world clinical settings for improving the health of individuals with NCDs who smoke.

The current intervention model may also protect the public from the health risks of second-hand smoke exposure by engaging hard-to-reach individuals in smoking cessation interventions, creating a smoke-free environment for future generations. Additionally, it could reduce the economic burden placed by smoking and related NCDs on the healthcare system. These outcomes will inform policies and guidelines regarding smoking cessation among individuals with no intention of quitting, thereby boosting sustainable development. Although the proportion of participants achieving smoking abstinence was relatively small (<5%), the current intervention model tripled the likelihood of biochemically validated smoking abstinence, increased intention to quit, and decreased cigarette consumption. The effect size (rate ratio) for the primary outcomes was encouraging, providing a foundation for mobile instant messaging-delivered brief MI, which may yield beneficial effects when implemented at the population level. Future research is warranted to evaluate and expand the effectiveness of this intervention in promoting smoking cessation among individuals who share other characteristics but have no intention to quit.

Advances in artificial intelligence (AI)-powered mobile instant messaging technology represent a paradigm shift in public health efforts, and its integration into smoking cessation services is inevitable^31^. Despite AI-powered mobile technology (e.g., chatbots) providing an alternative to offer personalised, real-time smoking cessation support, their capacity to engage individuals who smoke remains inconclusive^32^. Healthcare professionals play a vital role in the current intervention model, given that specific brief MI counselling techniques and professional clinical judgment were required to resolve strong ambivalence about quitting among individuals who had no intention to quit^16,17^. Despite the human-led intervention remains indispensable in the field of smoking cessation, especially for individuals with no intention of quitting, AI-powered mobile instant messaging tools could be considered as a complement to support smoking cessation efforts. Future research should consider developing and evaluating an appropriate human-AI blended care system that combines human-led therapeutic counselling with the scalability of AI.

## Methods

### Study design

An assessor-blinded, multicentre RCT was conducted at the outpatient clinics of three major acute care hospitals in Hong Kong. The methods and reporting followed the extension of the Consolidated Standards of Reporting Trials statement for social and psychological interventions (CONSORT-SPI 2018) (Supplementary Table 4). Ethical approval was obtained from the Institutional Review Board of the University of Hong Kong/Hospital Authority Hong Kong West Cluster, New Territories West Cluster Research Ethics Committee (REC), and Kowloon Central Cluster REC/Kowloon East Cluster REC. All participants provided written informed consent. This study was pre-registered on May 12, 2021 (ClinicalTrials.gov ID: NCT04890223).

### Participants

Eligible individuals who attended medical follow-ups in the selected outpatient clinics were invited to participate. According to the WHO, the major types of NCDs include cardiovascular diseases, cancers, chronic respiratory diseases, and diabetes^1^. The inclusion criteria were: (1) Hong Kong Chinese individuals aged over 17 who (2) had smoked at least one cigarette per day over the previous 3 months, (3) had been diagnosed with at least one NCD, (4) were able to speak Cantonese and read Chinese, (5) were willing to take action to improve their health but did not intend to quit smoking within 6 months, (6) had a smartphone and were able to use mobile instant messaging tools (i.e., WhatsApp or WeChat), and (7) were willing to receive health promotion advice and communicate via those tools. Individuals participating in other smoking cessation programs or services or with mental or cognitive impairment or communication problems were excluded.

### Randomisation and masking

Participants were randomised (1:1) to an intervention or control group using a set of computer-generated random numbers prepared by a research assistant not involved in participant recruitment. Randomly permuted block sizes of 4, 6, or 8 ensured similar numbers of participants in the two groups. The allocation sequence was concealed using sequentially numbered, opaque, sealed envelopes (SNOSE), which were opened by one of the research nurses (RNs) during group assignment after obtaining participants’ written informed consent and collecting their baseline data. The nature of behavioural intervention meant that it was not feasible to blind the participants or the RNs. Follow-up data were collected by research assistants blinded to group allocation.

### Procedures

The RNs identified eligible individuals by reviewing medical records and subsequently approached them to confirm eligibility. For those who met the criteria, the RNs provided study information, obtained written consent, and then opened the SNOSE. All interventions were conducted by trained RNs based on one unhealthy behaviour other than smoking, namely unhealthy diet, physical inactivity, unhealthy sleeping pattens, and excessive alcohol use, chosen by individual participants as the behaviour they most wished to change at baseline. The RNs had at least one year of experience conducting smoking cessation research and were trained by a clinical psychologist who is a member of the Motivational Interviewing Network of Trainers.

The intervention group received a 10-minute individual face-to-face brief MI session in the outpatient clinics, followed by individualised real-time brief MI messages through a mobile instant messaging tool for 6 months. The face-to-face brief MI sessions focused on reinforcing intrinsic motivation to change the selected unhealthy behaviour, assisting participants in developing change strategies, and consolidating change plan. The content of the brief MI messages was guided by the menu of strategies^25^ focused on four MI fundamental processes (engaging, focusing, evoking, and planning) and was tailored to the unhealthy behaviours, desired goal, progress in behaviour modification, and demographic characteristics (e.g., age, sex, and occupation) (Supplementary Table 5). The brief MI messages started with encouragement of the participants’ commitment to their change plans and proceeded to the provision of any requested assistance or information. The brief MI messages were then designed to encourage participants to explore further their current ambivalence and to think about the possibility of other behavioural change (i.e., quitting smoking) (Supplementary Fig. 2).

To maintain interactions with participants, brief MI messages were delivered to them twice a week to initiate conversations. A session of conversation served as the unit for measuring engagement with the intervention, and was considered to have ended after the participants had been absent from the conversation for an hour. Participants were free to initiate a conversation session at any time, though real-time responses from the RNs were only delivered during office hours (9 am to 7 pm). The RNs monitored the participants’ progress and assessed their willingness to take further steps toward behavioural change (i.e., expressing the intention to quit). After 6 months, only reminders for participating in the 12-month follow-up were delivered to maintain minimal contact. The research team members reviewed the conversation history regularly to ensure the intervention fidelity.

The control group received a 10-minute generic health advice consultation regarding the selected unhealthy behaviours, in addition to the usual clinical practice that included a self-help smoking cessation booklet along with a public quitline number. Three follow-up calls were made to conduct follow-up assessments and remind the participants of the next assessment. Those who expressed an intention to quit at the follow-ups were given information about available smoking cessation services, which is usual practice in clinical settings.

### Outcomes

Data were collected in-person at baseline and via telephone at 3, 6, and 12 months after baseline, with biochemical validation conducted in-person at 6 and 12 months. The primary outcome was biochemically validated smoking abstinence at 12 months, defined as an exhaled carbon monoxide level < 4 parts per million and a saliva cotinine level < 15 ng/mL^29^. Participants who self-reported abstinence within the past 7 days at the 6- and 12-month follow-ups were invited to attend the biochemical validation tests.

The secondary outcomes included biochemically validated smoking abstinence at 6 months, self-reported 7-day PPA, intention to quit, smoking reduction of at least 50% (from baseline), quit attempts, and changes in the chosen unhealthy behaviours, all of which were assessed at 3, 6, and 12 months (Supplementary Table 6).

Effective engagement with the intervention, defined as at least three messaging conversations with the researchers during the intervention period, was a crucial factor in the evaluation of intervention efficacy^33^. The participants’ engagement with the intervention was checked against the backup files of their chats.

### Statistical analysis

The sample size was estimated according to a previous RCT of a mobile messaging-based smoking cessation intervention, which reported a rate ratio of 2.4 for biochemically validated smoking abstinence at 6 months^34^. To achieve 80% power at a one-sided significance level of 2.5%, we estimated that at least 248 participants per group (496 in total) were needed. Considering an expected retention rate of approximately 70% at 12 months, the target sample size was at least 710 participants (355 per group).

All analyses were performed using IBM SPSS 28 (IBM Crop., Armonk, NY) with the significance level set at 0.05 (two-sided). The participants’ baseline characteristics and outcomes are summarised and presented using appropriate descriptive statistics. Our primary focus was on the event rates of the outcomes at each follow-up time point, with particular emphasis on the primary endpoint of biochemically validated smoking abstinence at 12 months, rather than the event rate changes over time. In contrast to modelling such rate changes, accommodating correlation structures is not necessary when comparing event rates at separate time points. A generalised linear model with a log binomial link function was used to compare the event rates of outcomes between the intervention and control groups at each follow-up time point. Holm’s multiplicity adjustment procedure was used to control the family-wise type I error rate to account for comparing the event rates separately. Following the commonly adopted practice in smoking cessation research^11^, the missing outcomes were imputed as failure in the primary analysis. To evaluate the robustness of the primary analysis results using the intention-to-treat approach (ITT), a sensitivity analysis was performed based on completer cases, with the expectation that the results would not be threatened by attrition bias under the condition of missing completely at random (MCAR)^35^. A logistic regression analysis was conducted to examine the association between engagement with the intervention and the primary outcome.

## Supplementary information

Supplementary information

## Data Availability

The data that support the findings of this study are available from the Food and Health Bureau, Hong Kong SAR but restrictions apply to the availability of these data, which were used under licence for the current study, and so are not publicly available. Data are however available from the authors upon reasonable request and with permission of the Food and Health Bureau, Hong Kong SAR.
