# Supplementary material for: Instant messaging-delivered brief motivational interviewing for noncommunicable disease patients with no intention to quit smoking
Source: NPJ Digit Med. 2026 Apr 11;9:440. doi: 10.1038/s41746-026-02578-6 (PMC13253850; doi:10.1038/s41746-026-02578-6)
Supplement: Supplementary file 1 — Supplementary information [file 41746_2026_2578_MOESM1_ESM.docx]

Supplementary information

**Supplementary Figure 1.** Theoretical framework

Smokers with NCD

No intention to quit

Willing to take action to promote health

Identified unfavourable behaviour

Foot-in-the-door technique

(Small initial request)

The RN allowed participants to select one unhealthy behaviour they most wanted to change as a first step

**Face-to-face Brief MI**

- Building rapport
- Setting goals and develop a change plan

**Brief MI via mobile instant messaging**

- Explore further their current ambivalence
- Encourage to think about the possibility of behavioural change (i.e., quitting smoking)

(Introducing a larger request)

↑ likelihood of achieving the first successful step to change unhealthy behaviour

↑ likelihood of taking further steps for their health

**Brief MI via mobile instant messaging**

- Advice on smoking
- Benefits of the decision to change
- Setting of quit date

Expressing the intention to quit

- Promote smoking cessation
- Achieving smoking abstinence
- Report behavioural change
- Making quit attempts
- Report smoking reduction of at least 50%

**Supplementary Figure 2.**
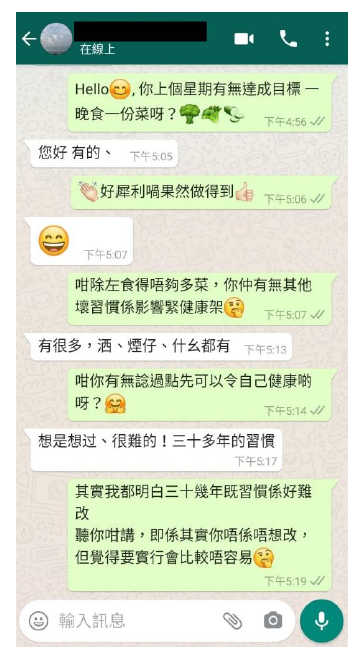
The content of brief MI messages

- The participant successfully met his goal of increasing vegetable intake. The RN acknowledged his effort and encouraged him to consider additional steps to further enhance his health.

**
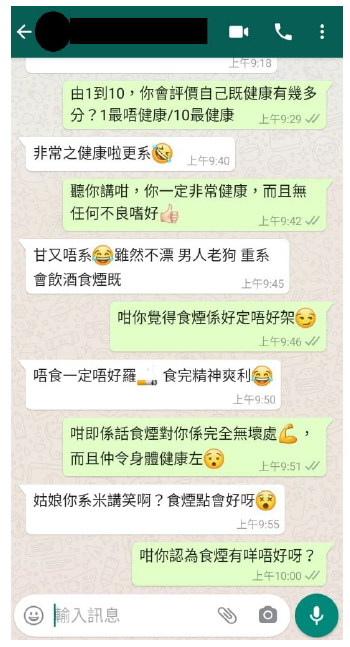
**

- The RN encouraged the participant to further reflect on his current situation and consider the possibility of making behavioural changes.


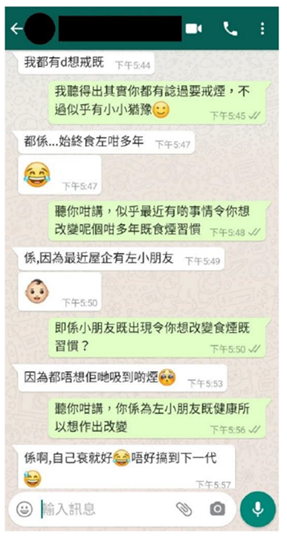


- The participant expressed a clear intention to reduce his daily cigarette consumption, which was recognised as change talk. The RN guided him in exploring his motivations and desire for change through reflective discussion.

**Supplementary Table 1.** Differences in the baseline characteristics by follow-up status at 12 months

|  |  | No. (%) |  | *P* value |
| --- | --- | --- | --- | --- |
|  |  | Completers  (n = 461) | Dropouts  (n = 267) |  |
| Age, mean (SD), y |  | 55.0 (12.3) | 53.7 (13.5) | 0.215 |
| Sex |  |  |  |  |
| Male |  | 430 (93.3) | 250 (93.6) | 0.851 |
| Female |  | 31 (6.7) | 17 (6.4) |  |
| Educational attainment |  |  |  |  |
| Primary or below |  | 108 (23.8) | 57 (21.5) | 0.064 |
| Secondary |  | 308 (67.8) | 171 (64.5) |  |
| Tertiary |  | 38 (8.4) | 37 (14.0) |  |
| Employment status |  |  |  |  |
| Employed |  | 294 (63.8) | 169 (63.3) | 0.897 |
| Unemployed |  | 167 (36.2) | 98 (36.7) |  |
| Diagnosis |  |  |  |  |
| Cardiovascular diseases |  | 97 (21.0) | 55 (20.7) | 0.767 |
| Cancer |  | 2 (0.4) | 1 (0.4) |  |
| Chronic respiratory diseases |  | 52 (11.3) | 30 (11.3) |  |
| Diabetes |  | 36 (7.8) | 14 (5.3) |  |
| Multiple chronic conditions * |  | 274 (59.4) | 166 (62.4) |  |
| Years of smoking ^†^ |  | 34.0 (13.6) | 32.6 (14.7) | 0.196 |
| Daily cigarette consumption |  |  |  |  |
| 1–10 |  | 144 (31.6) | 78 (29.3) | 0.569 |
| 11–20 |  | 162 (35.6) | 98 (36.8) |  |
| 21–30 |  | 114 (25.1) | 75 (28.2) |  |
| >30 |  | 35 (7.7) | 15 (5.6) |  |
| Nicotine dependence by the FTND |  |  |  |  |
| Mild, 0-3 |  | 59 (13.0) | 28 (10.8) | 0.680 |
| Moderate, 4-5 |  | 151 (33.2) | 87 (33.5) |  |
| Severe, 6-10 |  | 245 (53.8) | 145 (55.8) |  |
| Previous quit attempts |  |  |  |  |
| Yes (within 6 months) |  | 155 (33.9) | 70 (26.5) | 0.118 |
| Yes (beyond 6 months ago) |  | 202 (44.2) | 129 (48.9) |  |
| No |  | 100 (21.9) | 65 (24.6) |  |

Abbreviations: FTND, Fagerström Test for Nicotine Dependence.

* Multiple chronic conditions: two or more concurrent chronic diseases.

|  | **No. /Total No. (%)** |  | |
| --- | --- | --- | --- |
|  | Biochemically validated smoking abstinence at 12 months | ORs (95% CI) | *P* value |
| **Did not engage** | 2/179 (1.1) | 1 [Reference] |  |
| **Effectively engaged** | 13/185 (7.0) | 6.69 (1.49–30.08) | .013 |

**Supplementary Table 2.** Engagement analysis (N = 364)

Abbreviations: CI, Confidence interval; OR, odds ratio.

| **Message types** | **Message/Session Characteristics** | **Frequency** | **Average total exposure per participant**^a^ |
| --- | --- | --- | --- |
| **Pre-scripted MI Messages** | Average length: 2–3 sentences | 2x per week | 48 messages |
| **Real-time conversation sessions** | Average duration^b^: 17.4 minutes  (SD = ± 3.1)  Range: 11–24 minutes | / | 26 sessions (7.5 hours) |

**Supplementary Table 3.** Average engagement time for brief MI (N = 364)

^a^ Mean total amount of the intervention that each participant in the intervention study received

^b^ Average Duration = (Sum of the duration of all real-time sessions) ÷ (Total number of sessions)

**Supplementary Table 4.** CONSORT 2025 checklist

|  | Section/topic | No | CONSORT 2025 checklist item description | Reported on page no. |
| --- | --- | --- | --- | --- |
|  | **Title and abstract** | | |  |
|  | Title and structured abstract | 1a | Identification as a randomised trial | 1 (Title page) |
|  |  | 1b | Structured summary of the trial design, methods, results, and conclusions | 2 (Abstract) |
|  | **Open science** | | |  |
|  | Trial registration | 2 | Name of trial registry, identifying number (with URL) and date of registration | 2 (Abstract) |
|  | Protocol and statistical analysis plan | 3 | Where the trial protocol and statistical analysis plan can be accessed | 12, 16 (Methods) |
|  | Data sharing | 4 | Where and how the individual de-identified participant data (including data dictionary), statistical code and any other materials can be accessed | NA |
|  | Funding and conflicts of interest | 5a | Sources of funding and other support (eg, supply of drugs), and role of funders in the design, conduct, analysis and reporting of the trial | 17 (Role of the Funder/Sponsor) |
|  |  | 5b | Financial and other conflicts of interest of the manuscript authors | 17 (Role of the Funder/Sponsor) |
|  | **Introduction** | | |  |
|  | Background and rationale | 6 | Scientific background and rationale | 3-5 |
|  | Objectives | 7 | Specific objectives related to benefits and harms | 4-5 |
|  | **Methods** | | |  |
|  | Patient and public involvement | 8 | Details of patient or public involvement in the design, conduct and reporting of the trial | NA |
|  | Trial design | 9 | Description of trial design including type of trial (eg, parallel group, crossover), allocation ratio, and framework (eg, superiority, equivalence, non-inferiority, exploratory) | 11-12 (Study design) |
|  | Changes to trial protocol | 10 | Important changes to the trial after it commenced including any outcomes or analyses that were not prespecified, with reason | NA |
|  | Trial setting | 11 | Settings (eg, community, hospital) and locations (eg, countries, sites) where the trial was conducted | 11-12 |
|  | Eligibility criteria | 12a | Eligibility criteria for participants | 12 |
|  |  | 12b | If applicable, eligibility criteria for sites and for individuals delivering the interventions (eg, surgeons, physiotherapists) | NA |
|  | Intervention and comparator | 13 | Intervention and comparator with sufficient details to allow replication. If relevant, where additional materials describing the intervention and comparator (eg, intervention manual) can be accessed | 13-15 |
|  | Outcomes | 14 | Prespecified primary and secondary outcomes, including the specific measurement variable (eg, systolic blood pressure), analysis metric (eg, change from baseline, final value, time to event), method of aggregation (eg, median, proportion), and time point for each outcome | 15 |
|  | Harms | 15 | How harms were defined and assessed (eg, systematically, non-systematically) | NA |
|  | Sample size | 16a | How sample size was determined, including all assumptions supporting the sample size calculation | 16 |
|  |  | 16b | Explanation of any interim analyses and stopping guidelines | NA |
|  | Randomisation: |  |  |  |
|  | Sequence generation | 17a | Who generated the random allocation sequence and the method used | 12-13 |
|  |  | 17b | Type of randomisation and details of any restriction (eg, stratification, blocking and block size) | 12-13 |
|  |  |  |  | **Reported on page no.** |
|  | Allocation concealment mechanism | 18 | Mechanism used to implement the random allocation sequence (eg, central computer/telephone; sequentially numbered, opaque, sealed containers), describing any steps to conceal the sequence until interventions were assigned | 12-13 |
|  | Implementation | 19 | Whether the personnel who enrolled and those who assigned participants to the interventions had access to the random allocation sequence | 12-13 |
|  | Blinding | 20a | Who was blinded after assignment to interventions (eg, participants, care providers, outcome assessors, data analysts) | 12-13 |
|  |  | 20b | If blinded, how blinding was achieved and description of the similarity of interventions | 12-13 |
|  | Statistical methods | 21a | Statistical methods used to compare groups for primary and secondary outcomes, including harms | 16 |
|  |  | 21b | Definition of who is included in each analysis (eg, all randomised participants), and in which group | 16 |
|  |  | 21c | How missing data were handled in the analysis | 16 |
|  |  | 21d | Methods for any additional analyses (eg, subgroup and sensitivity analyses), distinguishing prespecified from post hoc | 16 |
|  | **Results** | | |  |
|  | Participant flow, including flow diagram | 22a | For each group, the numbers of participants who were randomly assigned, received intended intervention, and were analysed for the primary outcome | 6 & table 2 |
|  |  | 22b | For each group, losses and exclusions after randomisation, together with reasons | 5 & figure 1 |
|  | Recruitment | 23a | Dates defining the periods of recruitment and follow-up for outcomes of benefits and harms | 5 |
|  |  | 23b | If relevant, why the trial ended or was stopped | NA |
|  | Intervention and comparator delivery | 24a | Intervention and comparator as they were actually administered (eg, where appropriate, who delivered the intervention/comparator, how participants adhered, whether they were delivered as intended (fidelity)) | 5 & supplementary table 2, 3 |
|  |  | 24b | Concomitant care received during the trial for each group | NA |
|  | Baseline data | 25 | A table showing baseline demographic and clinical characteristics for each group | Table 1 |
|  | Numbers analysed,  outcomes and estimation | 26 | For each primary and secondary outcome, by group:  ● the number of participants included in the analysis  ● the number of participants with available data at the outcome time point  ● result for each group, and the estimated effect size and its precision (such as 95% confidence interval)  ● for binary outcomes, presentation of both absolute and relative effect size | 6, Figure 1 & table 2 |
|  | Harms | 27 | All harms or unintended events in each group | NA |
|  | Ancillary analyses | 28 | Any other analyses performed, including subgroup and sensitivity analyses, distinguishing pre-specified from post hoc | NA |
|  | **Discussion** | | |  |
|  | Interpretation | 29 | Interpretation consistent with results, balancing benefits and harms, and considering other relevant evidence | 7-11 |
|  | Limitations | 30 | Trial limitations, addressing sources of potential bias, imprecision, generalisability, and, if relevant, multiplicity of analyses | 9 |

Citation: Hopewell S, Chan AW, Collins GS, Hróbjartsson A, Moher D, Schulz KF, et al. CONSORT 2025 Statement: updated guideline for reporting randomised trials. BMJ. 2025; 388:e081123. <https://dx.doi.org/10.1136/bmj-2024-081123>
© 2025 Hopewell et al. This is an Open Access article distributed under the terms of the Creative Commons Attribution License (<https://creativecommons.org/licenses/by/4.0/>), which permits unrestricted use, distribution, and reproduction in any medium, provided the original work is properly cited.

*We strongly recommend reading this statement in conjunction with the CONSORT 2025 Explanation and Elaboration and/or the CONSORT 2025 Expanded Checklist for important clarifications on all the items. We also recommend reading relevant CONSORT extensions. See [www.consort-spirit.org](http://www.consort-spirit.org).

| **Core elements of the menu of strategies** | **Purposes** | **Examples of brief MI messages** |
| --- | --- | --- |
| Opening discussion | - To build trusting relationships with patients - To explore and understand patients’ perspectives, values, and beliefs regarding the behaviour change | *“Do you still have other behaviour that you consider unhealthy?”*  *“Can you tell me more about your smoking habit?”* |
| A typical day/my lessons | - To encourage patients to share their behaviour in greater detail - To identify triggers of or barriers to the behaviour | *“Can you describe how you start your day with cigarettes?”*  *“Tell me more how your smoking habit in relation to your job.”* |
| The good things and the less-good things | - To identify the good things and the less-good things associated with the behaviour - To encourage patients to explore their ambivalence about behavioural change - To increase intrinsic motivation to change by discussing the less-good things of the behaviour | *“What are the good things about continuing to smoke?”*  *What are the less-good things about quitting smoking?”* |
| Providing information | - To provide information only upon patients’ request - To share other patients’ experiences | *“I could offer you some information about strategies for quitting if you are interested.”* |
| The future and the present | - To explore the discrepancies between patients’ current and desired behaviours - To raise patients’ awareness of the negative consequences of the current behaviour | *“How would you like things to be different in the future?”*  *“How does your current behaviour differ from your desired behaviour?”* |
| Exploring concerns | - To encourage patients to identify their concerns about behavioural change - To offer advice upon requests | *“What concerns do you have about quitting?”*  *“What is stopping you from quitting?”* |
| Helping with decision making | - To elicit patients from deliberating their possible reason and desire for change - To increase their intrinsic motivation to change | *“How do you feel about this now?”*  *“What are you going to do about this now?”*  *“What is your plan now?”* |

**Supplementary Table 5.** The menu of strategies of brief MI and examples of brief MI messages

**Supplementary Table 6.** The operational definitions of the primary and secondary outcomes

| **Primary and secondary outcomes** | **Operational definitions** |
| --- | --- |
| 7-day point-prevalence abstinence | Complete abstinence for seven days prior to assessment |
| Intention to quit | Individual’s readiness to quit smoking within six months (i.e. were in the contemplation and preparation stages) |
| Smoking reduction of at least 50% | Reduction in cigarette consumption from the baseline by at least 50% |
| Quit attempts | A period of intentional abstinence of more than 24 hours |
| Changes in the chosen unhealthy behaviours | Individual’s perceived modification of the selected unhealthy behaviour |
